# Isotope-Assisted Metabolite Analysis Sheds Light on Central Carbon Metabolism of a Model Cellulolytic Bacterium *Clostridium thermocellum*

**DOI:** 10.3389/fmicb.2018.01947

**Published:** 2018-08-23

**Authors:** Wei Xiong, Jonathan Lo, Katherine J. Chou, Chao Wu, Lauren Magnusson, Tao Dong, PinChing Maness

**Affiliations:** National Renewable Energy Laboratory, Golden, CO, United States

**Keywords:** ^13^C-isotope tracer, cellulolytic bacteria, citrate synthase, glycolytic pathways, isoleucine biosynthesis, metabolic flux analysis, tricarboxylic acid cycle

## Abstract

Cellulolytic bacteria have the potential to perform lignocellulose hydrolysis and fermentation simultaneously. The metabolic pathways of these bacteria, therefore, require more comprehensive and quantitative understanding. Using isotope tracer, gas chromatography-mass spectrometry, and metabolic flux modeling, we decipher the metabolic network of *Clostridium thermocellum*, a model cellulolytic bacterium which represents as an attractive platform for conversion of lignocellulose to dedicated products. We uncover that the Embden–Meyerhof–Parnas (EMP) pathway is the predominant glycolytic route whereas the Entner–Doudoroff (ED) pathway and oxidative pentose phosphate pathway are inactive. We also observe that *C. thermocellum*'s TCA cycle is initiated by both *Si*- and *Re-*citrate synthase, and it is disconnected between 2-oxoglutarate and oxaloacetate in the oxidative direction; *C. thermocellum* uses a citramalate shunt to synthesize isoleucine; and both the one-carbon pathway and the malate shunt are highly active in this bacterium. To gain a quantitative understanding, we further formulate a fluxome map to quantify the metabolic fluxes through central metabolic pathways. This work represents the first global *in vivo* investigation of the principal carbon metabolism of *C. thermocellum*. Our results elucidate the unique structure of metabolic network in this cellulolytic bacterium and demonstrate the capability of isotope-assisted metabolite studies in understanding microbial metabolism of industrial interests.

## Introduction

Lignocellulosic biomass is the most abundant bio-renewable resource on earth. It has the potential to substitute the current sugar feedstocks for sustainable biofuels and biochemicals production. The use of lignocellulosic biomass has significant merits including improving net carbon and energy balances, reducing production cost, and avoiding the competition between food vs. fuel (Lynd et al., 2005, 2008). However, effective application of lignocellulose in bio-refineries is currently impeded by biomass recalcitrance: its inherent resistance to depolymerization. A potential solution is consolidated bioprocessing (CBP), in which microbes of outstanding cellulolytic ability are recruited for combined cellulose hydrolysis and fermentation without extra addition of cellulases (Lynd et al., 2005). *Clostridium thermocellum* is among the most attractive CBP host microbes.

*C. thermocellum* is a Gram-positive, thermophilic, and strict anaerobic bacterium. By taking advantage of an extracellular cellulase system called the cellulosome (Gold and Martin, 2007), *C. thermocellum* can degrade cellulose into soluble oligosaccharides. The latter can be further utilized by the cells and fermented to products of industrial interest (e.g., H_2_, formate, lactate, acetate, ethanol) (Ellis et al., 2012; Holwerda et al., 2014). Recent advances in genetic modification tools (Tripathi et al., 2010; Argyros et al., 2011) further enable heterologous expression of new functional pathways (e.g., pentose sugar utilization Xiong et al., 2018, isobutanol production Lin et al., 2015) into the bacterium, making this CBP bacterium an attractive and engineerable platform for biofuels or biochemicals production.

Applications of *C. thermocellum* in CBP require not only knowledge in molecular and genetic details of its cellulolytic system but also global understanding of its carbon metabolism. Detailing the carbon fluxes from metabolic pathways in *C. thermocellum* will guide design principles for efficient conversion of lignocellulosic sugars into dedicated products. To date, there have been a few published works describing the fermentation pathways in *C. thermocellum* (Zhang and Lynd, 2005; Lu et al., 2006; Nataf et al., 2010; Olson et al., 2010; Deng et al., 2013; Zhou et al., 2013), whereas metabolic characteristics of this species have not been comprehensively and quantitatively understood. Although the availability of genome sequence of *C. thermocellum* strains (DSM 1313 and ATCC 27405) has provided essential information and blue prints in terms of metabolic pathways, elaborate experimental validation is still required to fully realize its biotechnological applications.

Recently, ^13^C-tracer and metabolic flux analysis has emerged as a powerful tool for validating genome annotation and this technique has enabled us to identify a unique CO_2_-fixing one-carbon metabolic pathway in *C. thermocellum* (Xiong et al., 2016). Complementing classic biochemical approaches in understanding metabolism such as enzyme activity assay, gene mutagenesis and the use of specific inhibitors to key metabolic steps, it provides a uniquely non-invasive approach to delineate the cellular metabolism *in vivo*. Here we use ^13^C-labeled substrates as isotopic tracers to follow the operation of *C. thermocellum's* central carbon metabolism and other primary metabolic pathways directly in living cells. As a verification of genomic information, we find that the Embden–Meyerhof–Parnas (EMP) pathway is the predominant glycolytic route whereas the Entner–Doudoroff (ED) pathway and oxidative pentose phosphate pathway are inactive. We also observe that *C. thermocellum's* TCA cycle is initiated with both *Si*- and *Re*- citrate synthase and it is broken between 2-oxoglutarate and oxaloacetate in the oxidative direction. *C. thermocellum* uses a citramalate shunt to synthesize isoleucine; and the one-carbon metabolism and the malate shunt are active in this bacterium.

To gain a quantitative insight into the newly identified metabolic activities, we constructed a fluxomic model that enables us to quantify the metabolic fluxes through the EMP pathway, the nonoxidative pentose phosphate pathway, the TCA cycle, the malate shunt, and amino acid biosynthesis pathways. This study represents the first global characterization of the central carbon metabolic pathways in a model cellulolytic species. It also showcases the capability of isotope-assisted metabolite analysis in complementing genome annotation with a more in-depth understanding of microbial metabolism.

## Materials and methods

### Strains, culture conditions, and medium

*C. thermocellum* DSM 1313 derived strain Δ*hpt* (Xiong et al., 2016) was grown anaerobically at 55°C on 5 g/L glucose. We used the DSM122-defined medium referred to as CTFUD minimum medium (Olson and Lynd, 2012). The rich medium was added with 1 g/L yeast extract. The growth medium was deoxygenated by gassing with argon for 20 min and autoclaved before use.

### Quantitative analysis of fermentation products

Cell growth was measured as a function of optical density by spectrophotometry (DU800; Beckman Coulter) at OD_600nm_. An OD_600_ of 1 correlated to 1.04 g/L cell dry weight (*R*^2^ = 0.9918). Hydrogen gas was measured using a gas chromatograph (7890A GC; Agilent Technologies) equipped with a thermal conductivity detector and a stainless-steel Supelco 60/80 Mol Sieve column (6 ft × 1/8 in) with argon as the carrier gas. Peak areas were compared with a standard curve. Glucose, lactate, formate, acetate, and ethanol were measured by HPLC (1200 series; Agilent Technologies) with a mobile phase of 4 mM H_2_SO_4_ at 0.6 mL/min flow rate using an Aminex HPX-87H column with a Micro Guard Cation H Cartridge. The column temperature was set to 55°C.

### ^13^C-tracer experiment

We adopted a steady-state labeling strategy. The ^13^C-labeling experiment was performed with different nutrient combinations: (1) 5.56 mM [U-^13^C6] glucose (20%) + 22.22 mM unlabeled glucose (80%); (2) 20 mM ^13^C-bicarbonate + 27.78 mM (5 g/L) glucose; (3) 20 mM ^13^C-formate + 27.78 mM glucose. (4) 20 mM [U-^13^C5] glutamate + 27.78 mM glucose. These nutrient combinations are supplemented into the CTFUD defined medium, respectively. For cell growth, *C. thermocellum* strains were inoculated into these media with a starting OD_600_ of 0.05. When late log phase was reached (OD_600_ above 0.6), 3 mL of cultures was sampled.

### GC-MS and isotopomer analysis for proteinogenic amino acids

The sample treatment and GC-MS analysis were done as previously reported (5), with a few modifications. Briefly, 3 mL of sampled cultures was centrifuged at 10,000 × g for 1 min, and the cell pellets were digested with 500 μL of 6 M HCl at 105°C for 12 h. The hydrolysate was dried under nitrogen gas flow at 65°C and dissolved in 50 μL of water-free dimethylformamide. For the GC/MS measurement, the proteinogenic amino acids were derivatized before analysis. The dried hydrolysate, dissolved in N, N-dimethylformamide, was derivatized by 1% tert-butyl-dimethylchlorosilane (TBDMS) at 85°C for 60 min. One microliter of the sample in the organic phase was loaded onto the GC/MS instrument [Agilent GC-6890 gas chromatograph equipped with an Agilent 19091J-413 column (30 m × 0.32 mm × 0.25 μm) directly connected to a MS-5975C mass spectrometer]. Helium was the carrier gas. The oven temperature was initially held at 50°C for 2 min; it was then raised to 150°C at 5°C/min and held at that value for 2 min. Finally, it was raised to 320°C at 7°C/min and held at that final value for 2 min. Other settings included splitless and electron impact ionization at 70 eV. The amino acids, including alanine, aspartate, glutamate, glycine, isoleucine, leucine, methionine, phenylalanine, proline, serine, threonine, tyrosine, and valine, were separated and analyzed. Histidine was not detected due to its low abundance in cell biomass.

To analyze the isotope labeling pattern of amino acids, a mass isotopomer distribution vector, *MDV*_α_, was assigned according to Nanchen et al. (2007).

(1) $$\begin{array}{l} {\text{MDV}}_{\alpha }=\left[\begin{array}{c} \left(m_{0}\right)\\ \left(m_{1}\right)\\ \vdots \\ \left(m_{n}\right) \end{array}\right]\sum_{i=0}^{n}m_{i}=1 \end{array}$$

where *m*_0_ is the fractional abundance of molecules with mono-isotopic mass and *m*_*i*__>0_ is the abundance of fragments with heavier masses. The GC-MS data were corrected for the naturally occurring isotopes of oxygen (O), hydrogen (H), and carbon (C) atoms using a correction matrix (Equation 2) as described by Nanchen et al. (2007).

(2) $$\begin{array}{l} MDV_{\alpha }^{*}=C_{\text{corr},{\text{COH}}^{-1}}.MDV_{\alpha } \end{array}$$

where ${\text{MDV}}_{\alpha }^{*}$ is the corrected mass isotopomer distribution vector and ${\text{C}}_{\text{corr},\text{COH}}^{-1}$ is the correction matrix. According to Equation 3, the resulting ${\text{MDV}}_{\alpha }^{*}$ values were then used to assess the fractional labeling enrichment of amino acids, respectively.

(3) $$\begin{array}{l} FL=\frac{\sum_{i=0}^{n}i.mi}{n.\sum_{i=0}^{n}mi} \end{array}$$

where *n* represents the number of carbon atoms in the amino acid and *i* is the mass isotopomer.

### Quantification of metabolic fluxes

The central carbon network of *C. thermocellum* DSM 1313 was constructed based on genome knowledge which has been validated by isotope tracer experiments in this work. Specifically, the network includes the EMP pathway, the nonoxidative pentose phosphate pathway, the malate shunt, the incomplete TCA Cycle led by both *Si*- and *Re*-citrate synthase, and amino acids biosynthesis pathways (Complete reactions list please see Supplementary File 1). The biomass composition (Supplementary File 1, R53) was defined according to a previous report describing genome-scale reconstruction of *C. thermocellum* metabolic network (Roberts et al., 2010). Minor modifications include: (1) We formulate the biomass equation by using the metabolites appeared in the reactions list (Supplementary File 1). (2) Experimentally measured fatty acids profiles are employed (see Supplementary File 1). The ^13^C-metabolic fluxes are quantified by minimizing the sum-of-squared residuals (SSR) between computationally simulated and experimentally determined measurements. INCA, a ^13^C-flux software based on Matlab platform (Young, 2014) was utilized for flux estimation.

## Results

### Validation of the experimental system for ^13^C tracer analysis of *C. thermocellum*

The first task for ^13^C-tracer analysis is to validate the experimental system by examining the effect of medium nutrients on ^13^C-labeling. This is because complex carbon nutrients (i.e., amino acids in yeast extract) can be incorporated into cell biomass, and thus interfere with a quantitative flux analysis. To test medium effect, we used 20% fully ^13^C-labeled and 80% fully unlabeled glucose as the carbon source and cultivated the cells in CTFUD rich medium containing yeast extract (1 g/L) and CTFUD defined medium without yeast extract, respectively. We used cultures when cells were at late exponential phase of growth (OD_600_ above 0.6). The labeling steady state is assumed to be reached at this growth stage, we then quantify the fractional labeling of each proteinogenic amino acid (AA) fragments by GC-MS. As shown in Figure S1, the labeling ratio of most proteinogenic AA fragments in rich medium is far less than the theoretical value (20%), indicating that unlabeled amino acids in yeast extract were assimilated by *C. thermocellum* directly and thus diluting the labeling ratio significantly. In contrast, the labeling ratio of AA fragments in CTFUD define medium is much close to theoretical value (20%), confirming that ^13^C-labeling is not interfered by medium nutrients in the defined medium and the pseudo steady state for labeling has been reached. Overall, our results have shown that tracer experiment using minimal CTFUD medium is a valid system for ^13^C metabolic flux analysis.

### Key amino acids pathways

To examine amino acids biosynthetic pathways in *C. thermocellum*, the labeling profiles of 13 proteinogenic amino acids were analyzed. We used ^13^C-bicarbonate labeling data as the blueprint, since ^13^C-bicarbonate labeling leads to the [1-^13^C] pyruvate through reversed pyruvate ferredoxin oxidoreductase (PFOR) (Xiong et al., 2016), and pyruvate may serve as the precursor of several key amino acids. For example, valine and leucine exhibited similar carbon molecule-labeling pattern of pyruvate (see Figure S2) and the labeled carbons were shown on the first position of these two amino acids (carboxylic group). The finding supports that pyruvate is the common precursor of both valine and leucine in *C. thermocellum* (see Figure S2).

With respect to aspartate biosynthesis, we detected that 15% of aspartate has two labeled carbons (Figure S3). It is consistent with a strong activity of anaplerotic reactions (the malate shunt), which enable the combination of [1-^13^C] pyruvate and a ^13^C-bicarbonate to form a two-carbon labeled malate and then oxaloacetate, the precursor of aspartate. We further observed that threonine has identical labeling pattern as that of aspartate, confirming that its carbon skeleton is derived from oxaloacetate directly (Figure S3).

Next, we examine the biosynthetic pathway for isoleucine, which is a typical branch chain amino acid. Genomic information (https://biocyc.org) suggests that two possible pathways may contribute to the biosynthesis of isoleucine: the threonine pathway and the citramalate pathway (Please see Figure S4 for detailed pathways). These two routes may lead to differential features in isotopomer labeling, thus enabling us to evaluate their relative contribution accordingly. The pathway from threonine may generate two-carbon labeled isoleucine (see Figure S4), while the citramalate pathway leads to only one ^13^C-carbon on the first position of isoleucine. The measured Mass Distribution Vector (MDV) of isoleucine showed that main labeling of isoleucine is exactly located at the carboxylic group of isoleucine (see Figure S4 Ile M-15, and Ile M-85) and the labeling probability on another carbon atom is comparatively low (m1: 7% in Ile M-85, see Figure S4). Apparently, this data suggests that the citramalate pathway serve as the dominant pathway for isoleucine biosynthesis.

### Glycolytic fluxes are dominantly contributed by the EMP pathway

**Figure 1:**
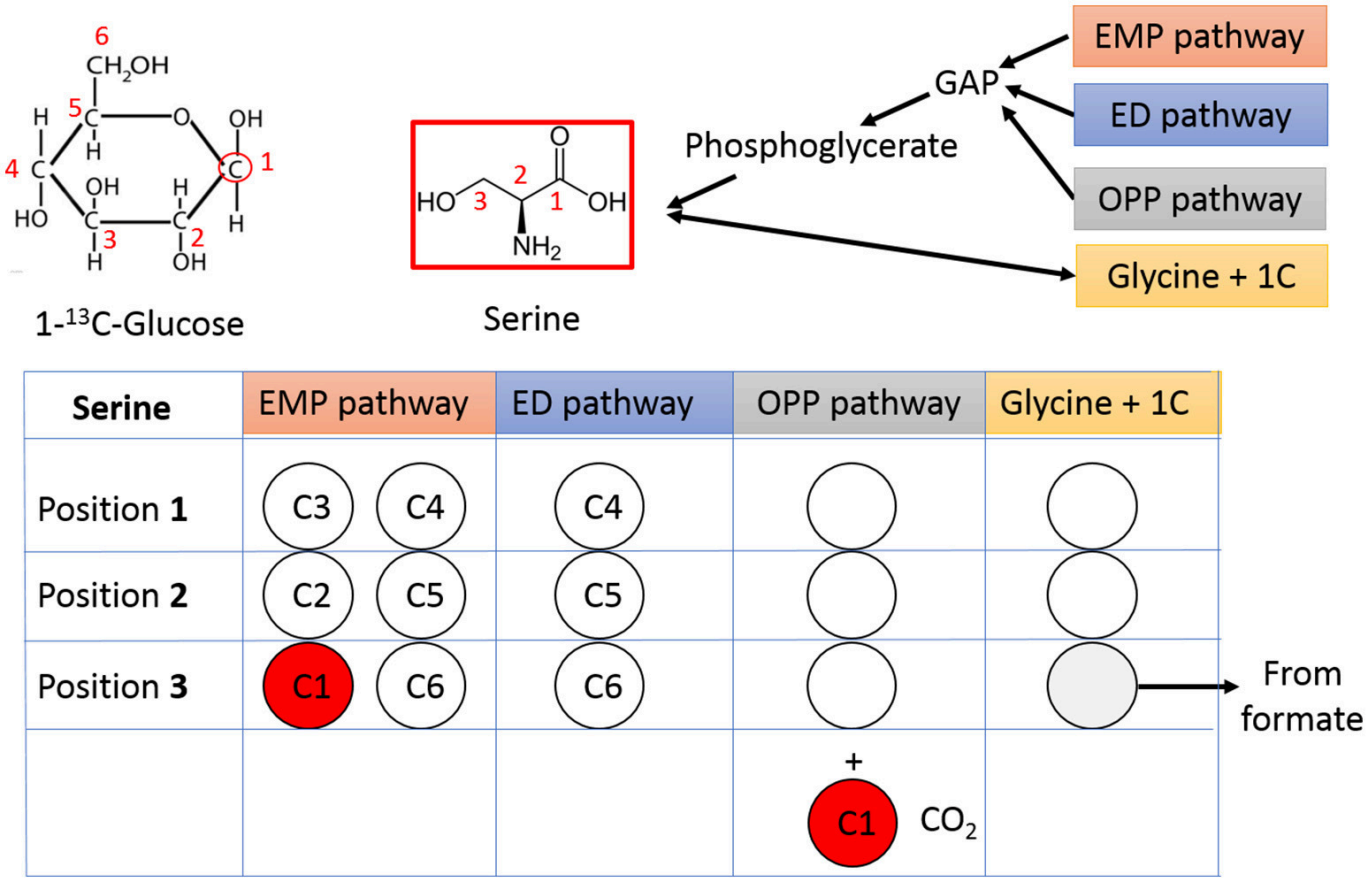
Tracing the glycolytic flux using 1-^13^C-glucose. Relative flux from different glycolytic pathways can be mirrored by the labeling pattern of serine. The unlabeled carbons are represented by open circles and labeled carbons are shown in red. The carbon number shown in each circle is from corresponding carbon in 1-^13^C-glucose. GAP, Glyceraldehyde 3-phosphate; OPP pathway, Oxidative Pentose Phosphate pathway.

**Figure 2:**
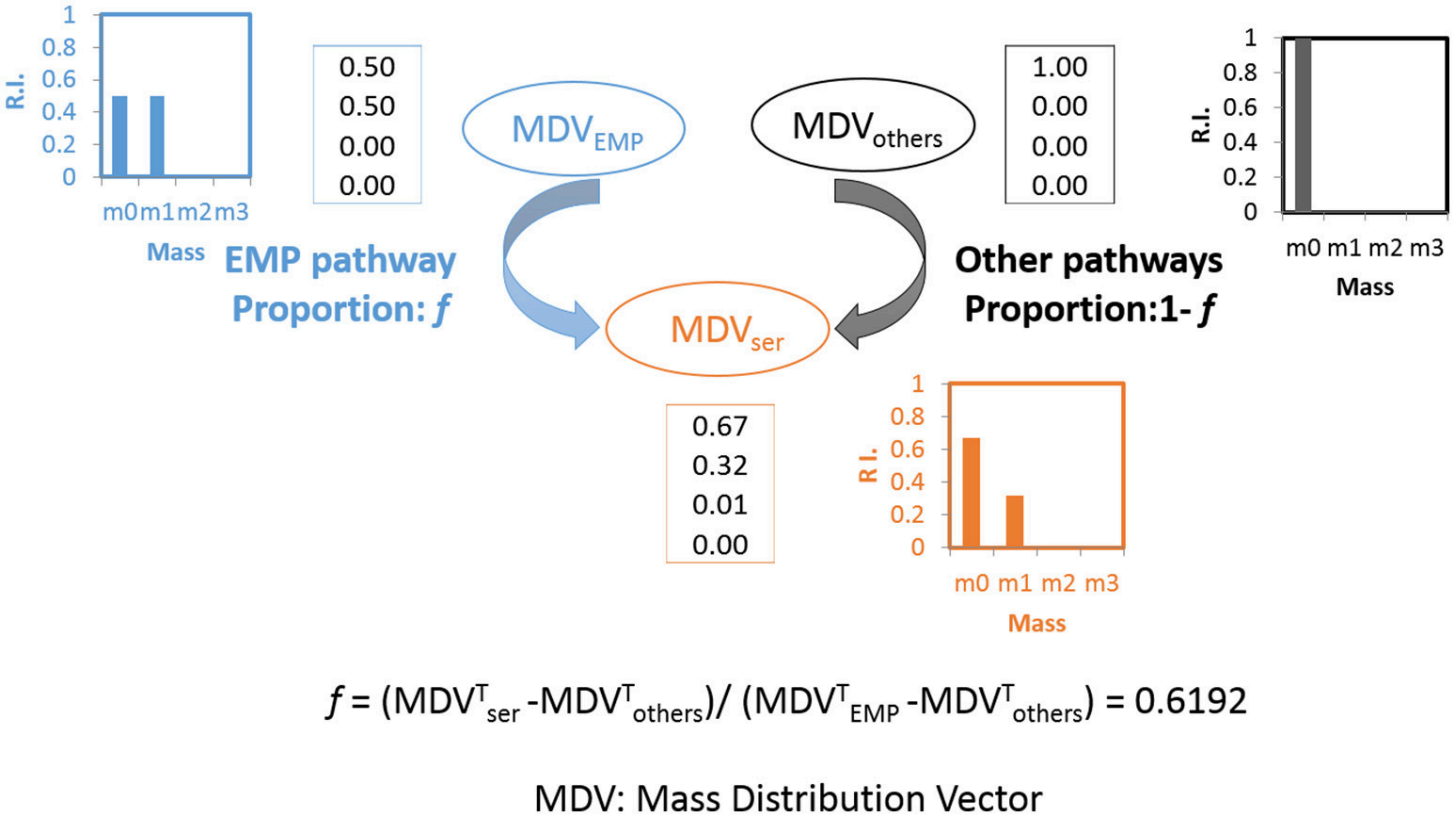
Calculation of flux ratio: Serine from the EMP pathway (*f*). Upon [1-^13^C] glucose labeling, the serine molecules originate through the EMP pathway will lead to half of the serine molecules labeled at position 3 of serine(m1: 0.5) while the other half will be unlabeled (m0: 0.5) (shown in blue). If the serine molecules originate from other pathways, none of the molecules will be labeled (m0: 1, shown in black). The final MDV of serine (shown in orange) is the cooperative result of these two possibilities and the splitting ratio (*f*) can be analyzed quantitatively. The algorithm is based on (Nanchen et al., 2007).

The pathway activities in *C. thermocellum* including the glycolytic pathway and the one-carbon pathway were then quantitatively analyzed by ^13^C-tracer analysis. Specifically, we utilized [1-^13^C] glucose as the carbon tracer to distinguish the glycolytic flux from the EMP pathway, the oxidative pentose phosphate pathway, and the ED pathway. Proteinogenic serine was used as the readout as it can be diversely synthesized from multiple routes of the glycolytic pathways. The deduced serine labeling pattern from each pathway is shown in Figure 1. Since the EMP pathway will split the hexose in the middle and results in 50% of serine labeled with one ^13^C atom, whereas other pathways only generate unlabeled serine (labeling ratio equals to 0%). The flux from the EMP pathway can be quantified accordingly and the results are shown in Figure 2. Our data show 62% of serine are derived from the EMP pathway, supporting that the EMP pathway is the dominant glycolytic route in *C. thermocellum*. However, origin of the rest 38% of serine remains as an intriguing question.

**Figure 3:**
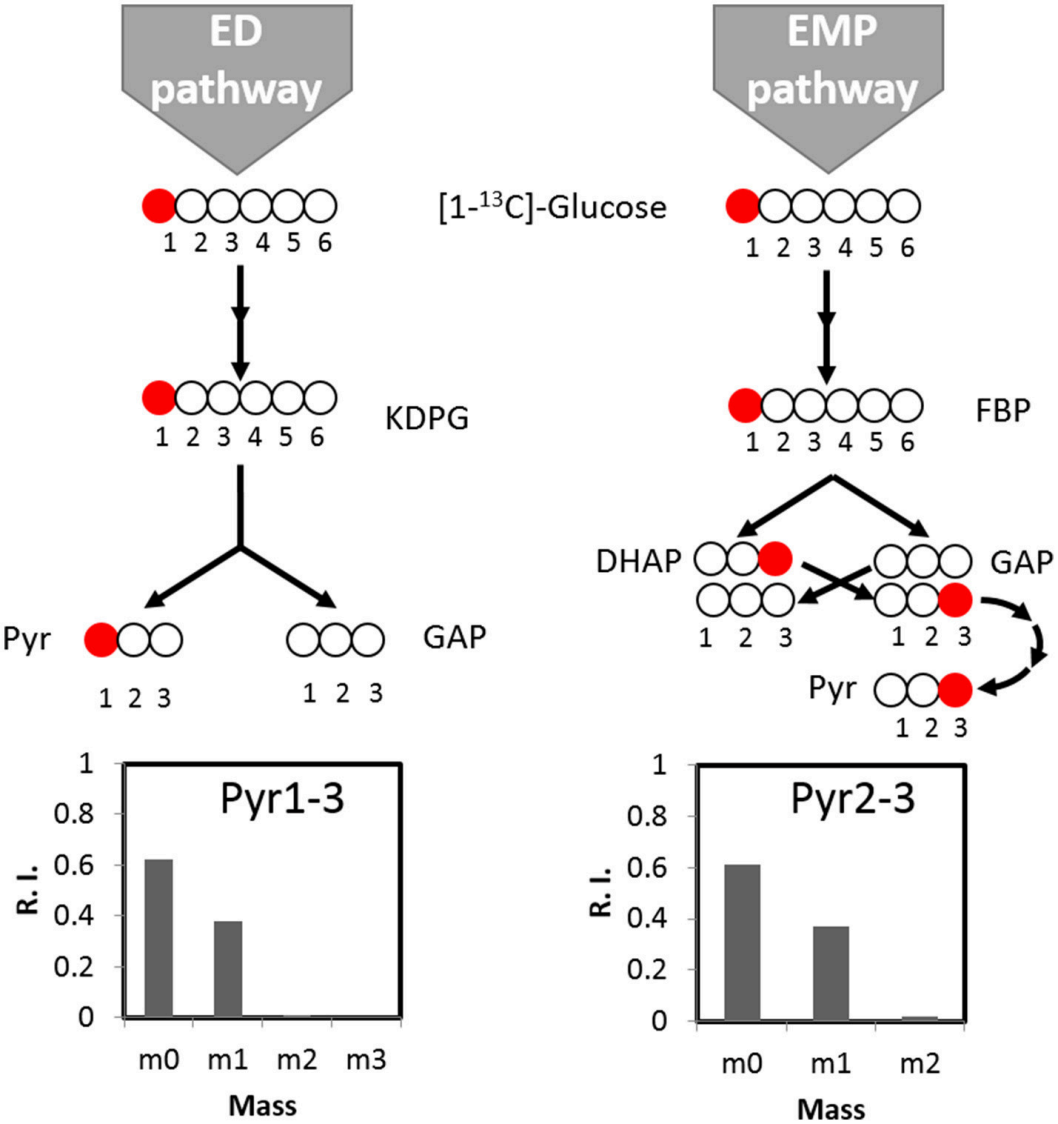
The activity of the ED pathway can be mirrored by pyruvate labeling pattern. If fluxes go through the ED pathway, [1-^13^C]glucose tracer will lead to pyruvate labeling at carboxylic group (C1). If fluxes go through the EMP pathway, pyruvate can be labeled at the C3 position. According to labeling pattern of alanine, the direct product of pyruvate, there is no significant labeling at C1 of pyruvate, indicating negligible activity of the ED pathway. KDPG, 2-keto-3-deoxygluconate-6-phosphate; FBP, Fructose 1,6-bisphosphate; DHAP, Dihydroxyacetone phosphate; GAP, glyceraldehyde 3-phosphate; Pyr, pyruvate.

We further tracked which other pathway(s) may contribute to the biosynthesis of the remaining labeled serine. With respect to the ED pathway, genes for ED pathway are not annotated in *C. thermocellum* genome but are identified in other *Clostridia* species (Bender and Gottschalk, 1973). The potential contribution from ED pathway therefore should be taken into account. We analyzed the ED pathway activity by checking the labeling pattern of alanine. If fluxes go through the ED pathway, [1-^13^C] glucose tracer will lead to labeling at the carboxylic group (C1) of pyruvate which is the precursor of alanine (Carbon destination of [1-^13^C] glucose by the ED pathway is shown in Figure 3). However, main labeling of alanine is found on C3 (Figure 3) identical to the EMP pathway activity, and negligible labeling is at C1 position. It clearly indicates very low activity of the ED pathway, which is consistent with the fact that key genes for ED pathway (i.e., 2-keto-3-deoxyphosphogluconate aldolase) is absent or not annotated based on the genomic information.

**Figure 4:**
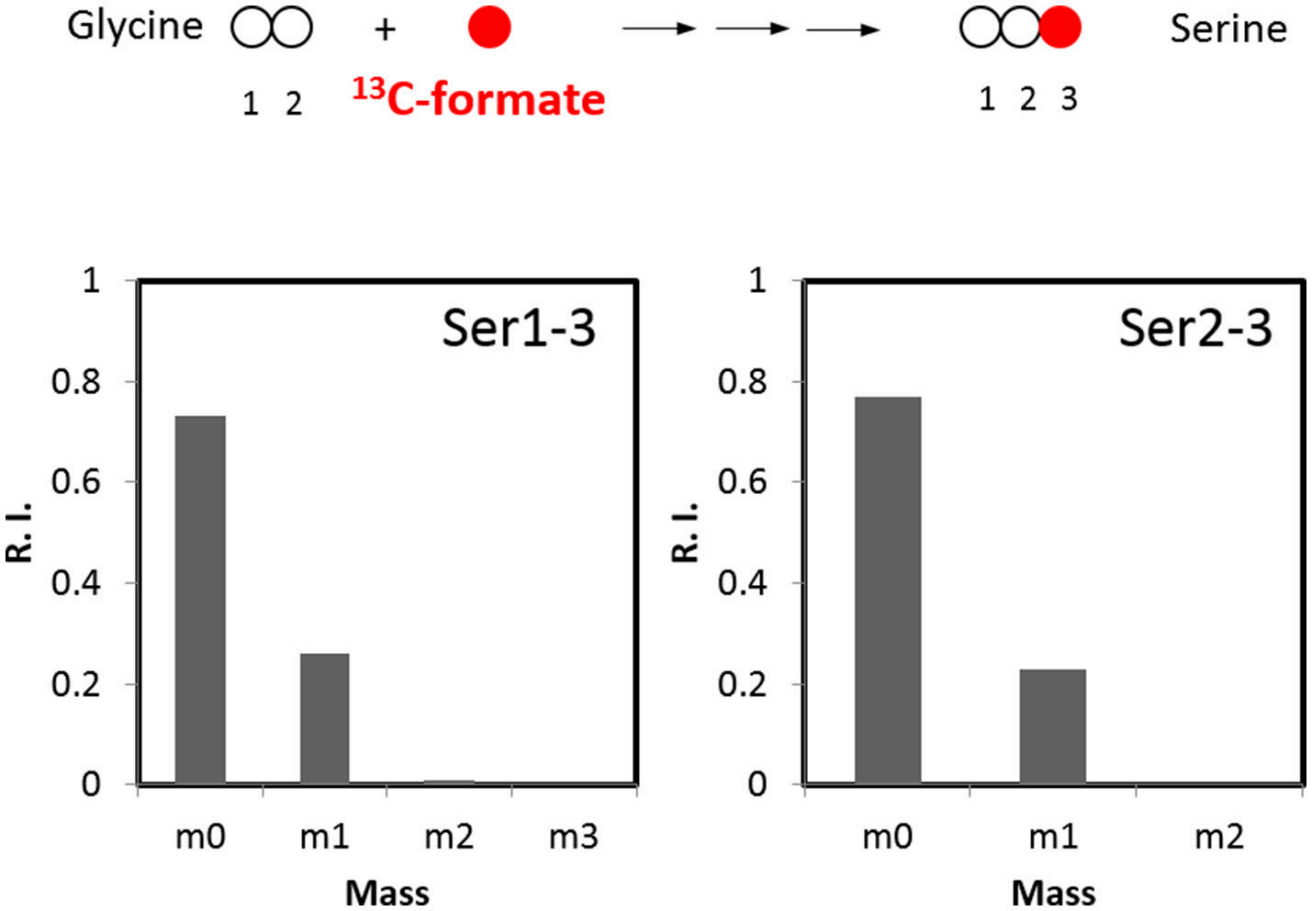
^13^C-formate tracer for analyzing the flux in the one-carbon pathway (*n* = 2, *SD* < 1%).

We next considered the activity of oxidative pentose phosphate pathway. Genes for the glucose 6-phosphate dehydrogenase and 6-phosphogluconate dehydrogenase are not found in *C. thermocellum* genome either. Additionally, their biochemical activity was absent from cell-free extracts (Patni and Alexander, 1971), making it unlikely that serine can be derived from this pathway. With respect to other pathways which may contribute to the biosynthesis of serine, we evaluated the importance of one-carbon metabolism. Serine can be reversibly synthesized from glycine by combining a methyl group which is derived from the formate via the one-carbon metabolism. To validate this pathway, we labeled the cells with ^13^C-formate and detected ~25% of serine labeled at C3 position (Figure 4). This result suggests the flux through one-carbon metabolism is quite high and dramatically contributes to serine synthesis. Overall, this analysis quantitatively informs us of the relative activities of glycolytic pathways and one-carbon metabolism.

### *C. thermocellum* carries both *Re*- and *Si*-citrate synthase

**Figure 5:**
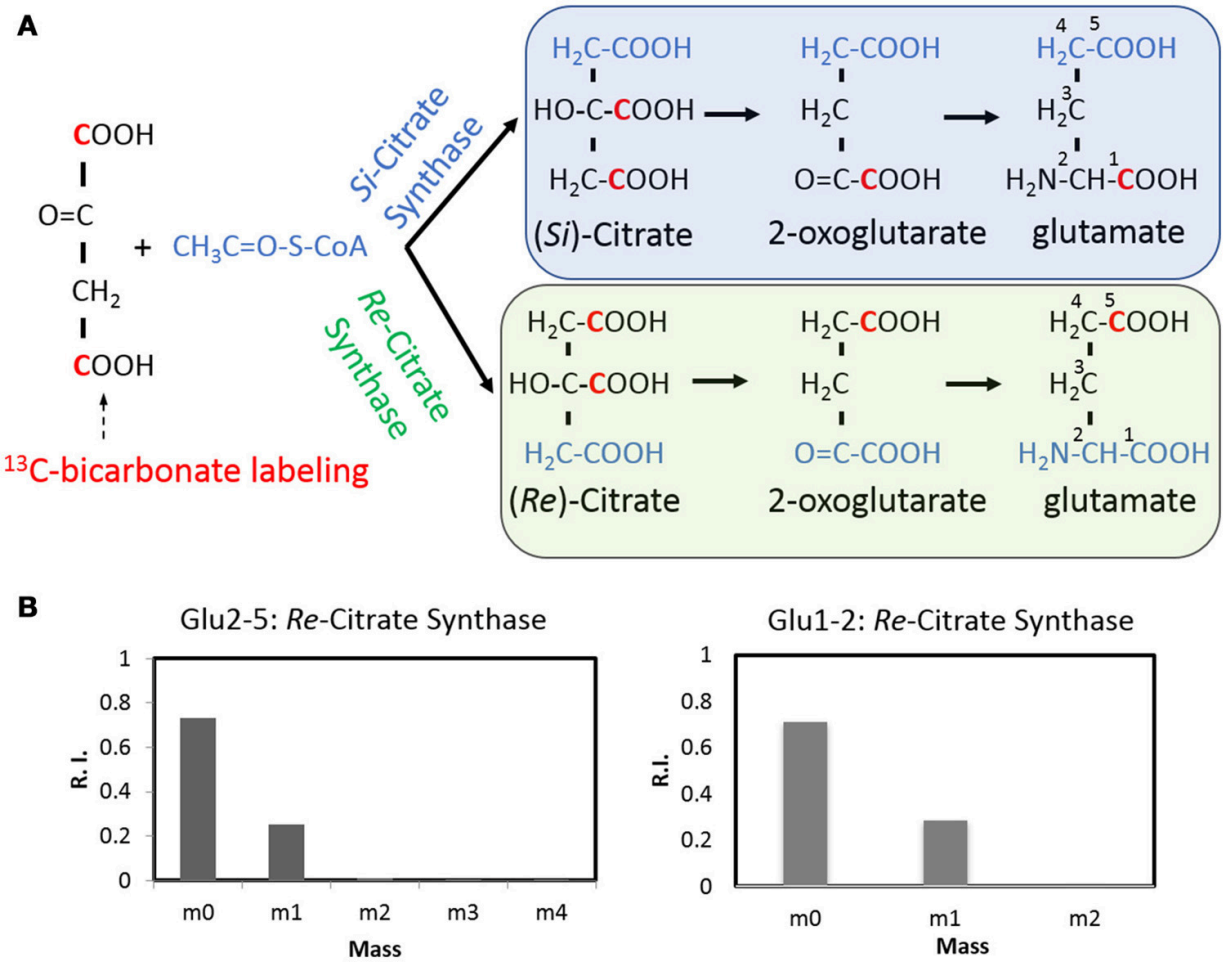
The stereospecificity of citrate synthase revealed by a ^13^C-bicarbonate labeling experiment **(A)** using GC-MS fragments Glu2-5 and Glu1-2 as the readouts **(B)**. The Re- citrate synthase will result in the labeling at the C5, thus consistent with one carbon labeled Glu2-5 fragment. The Si- citrate synthase activity will result in the labeling at the C1 position and lead to one carbon labeled Glu1-2 fragment.

As a ubiquitous energetic and biosynthetic pathway, the tricarboxylic acid (TCA) cycle however remains elusive for anaerobic bacteria. As the first step of the TCA cycle, biosynthesis of citrate from acetyl-coenzyme A and oxaloacetate is catalyzed in most organisms by a *Si-*citrate synthase, which is *Si*-face stereospecific with respect to C-2 of oxaloacetate. Whereas in *C. kluyveri* and some other clostridia, the reaction can be catalyzed by a Re-citrate synthase whose homolog was also found in *C. thermocellum* (Li et al., 2007). ^13^C tracer and isotopomer analysis offers an opportunity to visualize the stereospecificity of citrate synthase, *in vivo* (Wu et al., 2010). With respect to *C. thermocellum*, the stereospecificity of citrate synthase can be deduced by a ^13^C-bicarbonate labeling experiment (Figure 5) in which ^13^C-carbons can be propagated to both carboxylic group of oxaloacetate via the reactions of reversed PFOR (rPFOR) and malate shunt, and then to citrate by the stereospecific citrate synthases. The stereotype of citrate synthase can be mirrored by the labeling patterns of downstream glutamate (GC-MS fragments C2-5 and C1-2, respectively). As shown in Figure 5B, over 25% C2-5 fragment has one ^13^C-carbon, presumably on the δ-carboxylic group of glutamate. This is consistent with *Re*-type citrate synthase. We then checked the labeling pattern of glutamate C1-2. Interestingly 28% glutamate C1-2 fragment also has one ^13^C-carbon, suggesting the first carboxyl group of glutamate was labeled as well. This labeling pattern is consistent with the notion that *C. thermocellum* also carries an active *Si-*citrate synthase. To confirm this observation, we investigated the genome of *C. thermocellum* DSM 1313 and indeed the gene for *Si-*citrate synthase (CLO1313_RS02945) is present. It has 67% sequence identity with a known Clostridial *Si-*citrate synthase (Li et al., 2007). For *Re-*citrate synthase, we did the BLAST analysis using the sequence of a biochemically verified gene from *C. kluyveri* DSM 555 (Li et al., 2007). A candidate gene (CLO1313_RS03665) shares 61% identity with *C. kluyveri Re-* citrate synthase and a neighbor gene (CLO1313_RS03670) shares 73% homology with aconitase, strongly indicating their joint functionality in the TCA cycle by converting acetyl CoA and oxaloacetate to isocitrate. Integrating all the acquired knowledge, our data provides labeling evidence for the first time to support that *C. thermocellum* carries both *Re-* and *Si-*Citrate synthases to initiate the TCA cycle.

### The TCA cycle of *C. thermocellum* is incomplete

**Figure 6:**
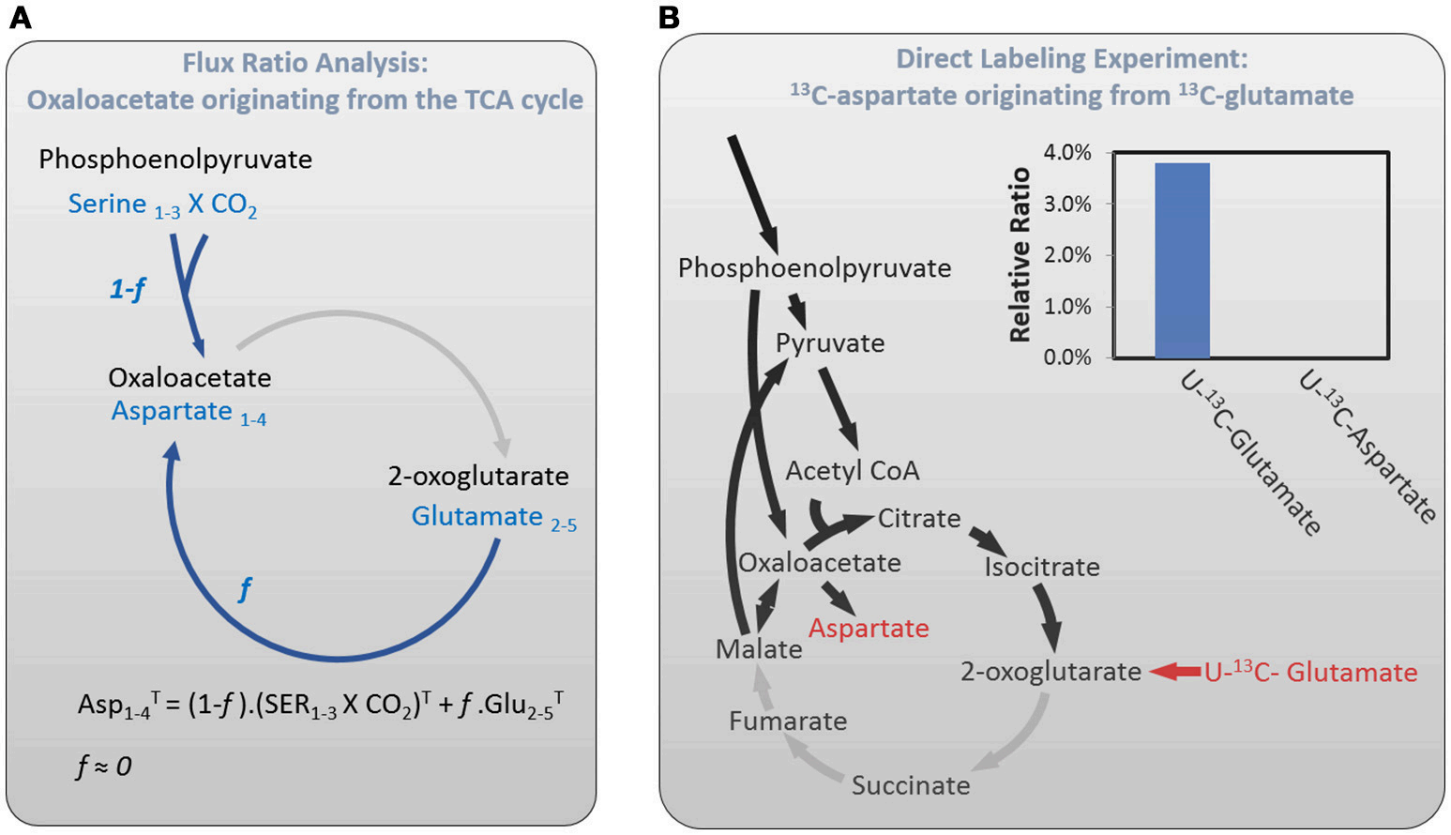
The metabolic flux from 2-oxoglutarate to oxaloacetate in oxidative direction analyzed by flux ratio approach using 20% [U-^13^C6] glucose plus 80% unlabeled glucose **(A)** and pulse labeling experiment using [U-^13^C5] glutamate as the tracer **(B)**.

It is generally accepted that most obligatory anaerobes do not harbor a complete oxidative TCA cycle, as energy can be acquired mostly from anaerobic fermentation. Recent studies however showed that a few model anaerobic bacteria such as *Proteus mirabilis* (Alteri et al., 2012) and *C. acetobutylicum* (Amador-Noguez et al., 2010) harbor a complete TCA cycle, even if genome annotation suggests the absence of certain TCA cycle enzymes. It is therefore worthwhile to decipher the structure of the TCA cycle in *C. thermocellum* specifically. First, we adopted the flux ratio analysis (Nanchen et al., 2007) to probe the completeness of the TCA cycle. Using [U-^13^C6] glucose experiments, we quantified the fraction of oxaloacetate originated from 2-oxoglutarate via the TCA cycle (*f*) vs. the ratio of oxaloacetate derived through anaplerotic reaction by e.g., phosphoenolpyruvate (PEP) carboxylase (1-*f*) (The algorithm please, see Figure 6A and Nanchen et al., 2007). Our calculation returned a *f* ≈ 0, representing the least squares solution of the relative flux from oxidative TCA cycle. This result indicates that the oxaloacetate is mainly synthesized by the anaplerotic reactions, whereas the flux barely reaches the oxaloacetate from the TCA cycle in the oxidative direction.

To further validate this calculation, we performed an additional pulse labeling experiment using [U-^13^C5] glutamate as the isotope tracer. After 12 h of labeling, we analyzed the proteinogenic glutamate and aspartate, respectively, which are produced from the TCA cycle (Figure 6B). Our result shows that nearly 4% fully labeled glutamate can be detected from the final glutamate pool, suggesting that the ^13^C-tracer was incorporated into the metabolic pathway. In comparison, no fully labeled aspartate was detectable during the labeling period. It indicates that the ^13^C carbons entering the 2-oxoglutarate branch cannot be propagated to oxaloacetate, the precursor of aspartate. Indeed, these fluxomic evidences are consistent with the genomic data that the absence of a few key enzymes including 2-oxoglutarate decarboxylase, fumarate reductase, and fumarase prevents *C. thermocellum* from forming a complete TCA cycle.

### Quantitative flux mapping

*In vivo* biochemical reaction rates are among the most important metabolic phenotypes. To obtain a quantitative insight into the fluxes in *C. thermocellum*, we developed a metabolic model that describes the isotope labeling of metabolites upon the addition of 20% universally labeled ^13^C-glucose and 80% unlabeled glucose (see section Materials and Methods, Figure S5 and Supplementary File 1 for modeling details). The biochemical reactions adopted in the model are based on genomic information and involve newly validated pathways that have been identified by isotope tracer experiments as described above. Specifically, the principal metabolic network includes the EMP pathway, anaplerotic reactions (malate shunt), citramalate pathway for isoleucine biosynthesis, *Re*- and *Si*-citrate synthases, incomplete TCA cycle, all of which have been verified by isotope tracer experiments in this work. In addition, we excluded the oxidative pentose phosphate pathway and the ED pathway from the central carbon metabolism which have been shown absent in *C. thermocellum* as described above. The balancing of energetic co-factors such as NAD(P)H is not input into the model either, because whether a particular redox reaction involves NADH vs. NADPH needs to be validated experimentally, and any assumptions for NAD(P)H-specific reactions will bring uncertainty to the modeling. Experimental inputs to the model include steady-state labeling data from proteinogenic amino acids, specific growth rates, sugar uptake rates, excretion rates for fermentation products (see Table S1), and flux ratio data at certain branch points: e.g., the relative contribution of the EMP pathway *vs*. one-carbon metabolism to serine biosynthesis as described above and the relative malate shunt and pyruvate phosphate dikinase fluxes to pyruvate, which has been quantitatively addressed recently (Olson et al., 2017).

**Figure 7:**
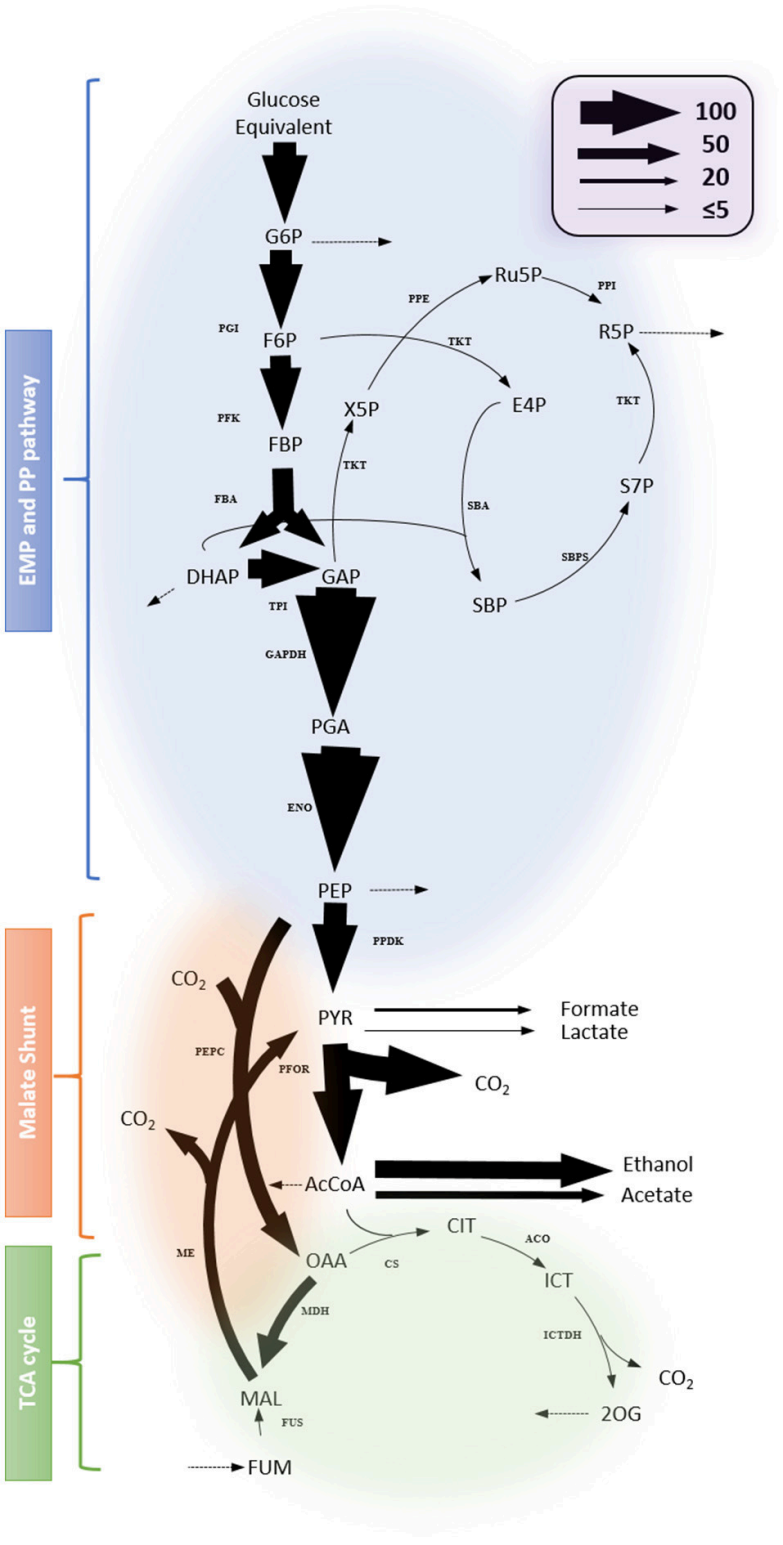
Flux map profiled from ^13^C-tracer experiment. Net fluxes are represented by proportionally-scaled arrow thickness and are normalized to a glucose monomer consumption rate of 100 (3.62 ± 0.18 mmol/gDW/h). Dotted arrows indicate fluxes toward biomass synthesis. Complete results including flux values with the 95% flux confidence interval are listed in Supplementary File 1. Abbreviations for metabolites: G6P, Glucose 6-phosphate; Ru5P, Ribulose 5-phosphate; RuBP, Ribulose bisphosphate; F6P, fructose 6-phosphate; R5P, Ribose 5-phosphate; FBP, Fructose bisphosphate; X5P, Xylulose 5-phosphate; E4P, Erythrose 4-phosphate; DHAP, Dihydroxyacetone phosphate; GAP, Glyceraldehyde-3-phosphate; SBP, Sedoheptulose bisphosphate; S7P, Sedoheptulose 7-phosphate; PGA, Phosphoglycerate; PEP, Phosphoenolpyruvate; PYR, pyruvate; AcCoA, Acetyl Coenzyme A; CIT, Citrate; ICT, Isocitrate; 2OG, 2-oxoglutarate; FUM, Fumarate; MAL, Malate; OAA, Oxaloacetate. Abbreviations for reactions: ACO, Aconitase; CS, citrate synthase; ENO, Enolase; FBA, Fructose bisphosphate aldolase; FUS, Fumarase; ICTDH, Isocitrate dehydrogenase; MDH, Malate dehydrogenase; ME, malic enzyme; PDH, pyruvate dehydrogenase; PEPC, Phosphoenolpyruvate carboxylase; PFK, Phosphofructosekinase; PFOR, pyruvate: ferredoxin oxidoreductase; PGI, Phosphoglucose isomerase; PK, pyruvate kinase; PPDK, Pyruvate phosphate dikinase; PRK, Phosphoribulokinase; PPE, Phosphopentose epimerase; PPI, Pentose phosphate isomerase; TAL, transaldolase;TKT, Transketolase; TPI, Triosephosphate isomerase; SBA, Sedoheptulose bisphosphate aldolase; SBPS, Sedoheptulose bisphosphatase.

Figure 7 shows representative results for the fitting data and a map of the identified flux values in central metabolism. The complete results are presented in Supplementary File 1 and the goodness for fitting in the Figure S5. The metabolic model fits the observed data. Most of the identified fluxes were tightly constrained, indicating that they reliably mirror the available experimental data. The results show that glycolytic flux predominates and is directed primarily toward the fermentation of lactate, formate, acetate and ethanol. Other apparent fluxes include aspartate production via malate shunt, and pentose-phosphate production from glycolytic intermediates. Within the incomplete TCA cycle, the flux through the oxidative branch is limited. The production of glutamate from citrate can occur via *Re*-citrate synthase but is also expected to occur via the *Si*-citrate synthase. Compared with the flux map of other *Clostridia* e.g., *C. acetobutylicum* (Amador-Noguez et al., 2010), the computational results presented herein demonstrate a featured fluxomic structure unique in *C. thermocellum*.

## Discussion

Using ^13^C-tracer studies, we have developed a quantitative flux model outlining the central carbon metabolism in *C. thermocellum* and elucidated the key pathways responsible for carbon flux distribution. This model could serve as a platform to probe cellular phenotypes upon subjecting to different growth conditions to further refine and validate the model and guide the genetic engineering strategies toward targeted products.

Versatile glycolytic pathways including the EMP pathway (Amador-Noguez et al., 2010), the ED pathway (Bender and Gottschalk, 1973), and the phosphoketolase pathway (Liu et al., 2012) have been found in *Clostridia*. These glycolytic pathways vary in reaction schemes and how much ATP and NAD(P)H they produce per glucose metabolized. Specific to *C. thermocellum*, however, we only identified the EMP pathway as the dominant one. Given various energetic and carbon requirements by the cells, *C. thermocellum* uses the EMP pathway exclusively, representing a typical metabolic rigidity. It implies that *C. thermocellum* may choose alternative strategies to modulate anaerobic energy and carbon demand. Indeed, the EMP pathway in *C. thermocellum* has many atypical features. For instance, the conversion of PEP to pyruvate features the malate shunt, which is believed to catalyze a transhydrogenase reaction converting NADH to NADPH and generate GTP (Deng et al., 2013). The flux through the malate shunt is regulated by ${\text{NH}}_{4}^{+}$ and pyrophosphate (PPi), which may indicate the metabolic state of the cell (Taillefer et al., 2015). Additionally, a number of phosphorylating enzymes in the EMP pathway rely on GTP/GDP/PPi, rather than ATP/ADP, including glucokinase and phosphoglycerate kinase (Zhou et al., 2013). Outside of glycolysis, *C. thermocellum* contains numerous enzymes that interconvert reduced ferredoxin and NAD(P)^+^ and influence energy metabolism including ferredoxin:NAD^+^ oxidoreductase (Rnf)(Müller et al., 2008), NADH-ferredoxin: NADP^+^ oxidoreductase (Nfn)(Wang et al., 2010), and hydrogenases. Understanding the metabolic network constrained by bioenergetic cofactors could help guide genetic strategies for strain redesign and optimization to ensure redox and energy balance to maximize the production of targeted products.

Our labeling results confirmed that *C. thermocellum* has activities for both *Si*- and *Re-*citrate synthase (Figure 5). Similar result was also observed in *C. kluyveri* (Li et al., 2007). So far, why two stereotypes of citrate synthase are contained in *Clostridia* remains an open question. *Si*- and *Re*-citrate synthase have no significant sequence homology (Li et al., 2007). Long phylogenetic distance indicates that they could be originated divergently. In addition, it should be noted that *Re*-citrate synthase is oxygen sensitive while *Si*-citrate synthase is not (Li et al., 2007). In view of citrate synthase's importance in initiating TCA cycle and in glutamate biosynthesis, the presence of two versions of citrate synthase could increase the sustainability of *C. thermocellum* in evolution, especially when subjecting to varying O_2_ atmosphere.

Our isotope experiment also demonstrates an incomplete TCA cycle in the oxidative direction from 2-oxoglutarate to oxaloacetate. This observation further prompts the question whether the TCA cycle in *C. thermocellum* can be operated reversibly in the reductive direction. In fact, the isotope data has excluded this possibility. In ^13^C-bicarbonate labeling experiment, we detected two-carbon labeled oxaloacetate. If it follows reductive operation of the TCA cycle, then 3-carbon labeled glutamate should be visualized due to one more ^13^C incorporation into the 2-oxoglutarate from the 2-oxoglutarate synthase. However, this is not observed in the glutamate labeling pattern (Figure 5), indicating no activity for the rTCA cycle. *C. thermocellum's* TCA cycle is different from that of *C. acetobutylicum*, the latter showed a complete, albeit bifurcated structure (Amador-Noguez et al., 2010). Our findings strengthen the notion that TCA cycle in anaerobes plays a major role in biosynthesis rather than bioenergetics, although the architecture of the cycle is species-specific.

The approach used in this study represents a standard and applicable methodology for metabolic flux analysis using steady-state isotope tracer experiments. The isotope labeling approach (steady-state isotopic approach) we used is different from the kinetic flux profiling which is another promising fluxomics method (Yuan et al., 2008). One major merit of steady-state isotopic approach is its capability in providing accurate ratios of fluxes at branch points. Additionally, only a few data points are needed for the analysis, satisfying the requirements for high-throughput and large-scale fluxomic analysis (Fischer et al., 2004; Fischer and Sauer, 2005). Other advantages include easy experimental procedures, simple modeling algorithm. Nevertheless, steady-state isotopic approach still has its limitation: e.g., it cannot discriminate the fluxes from multiple parallel pathways if no distinguishable labeling patterns can be generated. In the case of *C. thermocellum*, for example, such a scenario occurs in deciphering fluxes from pyruvate phosphate dikinase (*ppdk*) vs. malate shunt, both of which synthesize pyruvate from PEP but without forming distinguishable carbon-carbon bond. In this case, kinetic flux profiling which records labeling trajectories of intermediate as a function of time can provide a complementary solution (Olson et al., 2017). Joint and appropriate utilization of steady-state and kinetic fluxomics technique will enable a comprehensive understanding of the metabolic network by rationally designed isotope tracer experiments.

In conclusion, this work represents the first global investigation of the central carbon metabolism in *C. thermocellum* and exemplifies the ability of isotope tracer experiments and metabolic modeling in understanding microbial metabolism of industrial interests.

## Author contributions

WX and PM led the research. WX designed the experiments. WX, KC, CW, LM, and TD performed the experiments. WX, JL, KC, and PM wrote the article.

### Conflict of interest statement

The authors declare that the research was conducted in the absence of any commercial or financial relationships that could be construed as a potential conflict of interest.
