# Supplementary material for: Isotope-Assisted Metabolite Analysis Sheds Light on Central Carbon Metabolism of a Model Cellulolytic Bacterium Clostridium thermocellum
Source: Front Microbiol. 2018 Aug 23;9:1947. doi: 10.3389/fmicb.2018.01947 (PMC6115520; doi:10.3389/fmicb.2018.01947)
Supplement: Supplementary file 2 [file Data_Sheet_1.PDF]

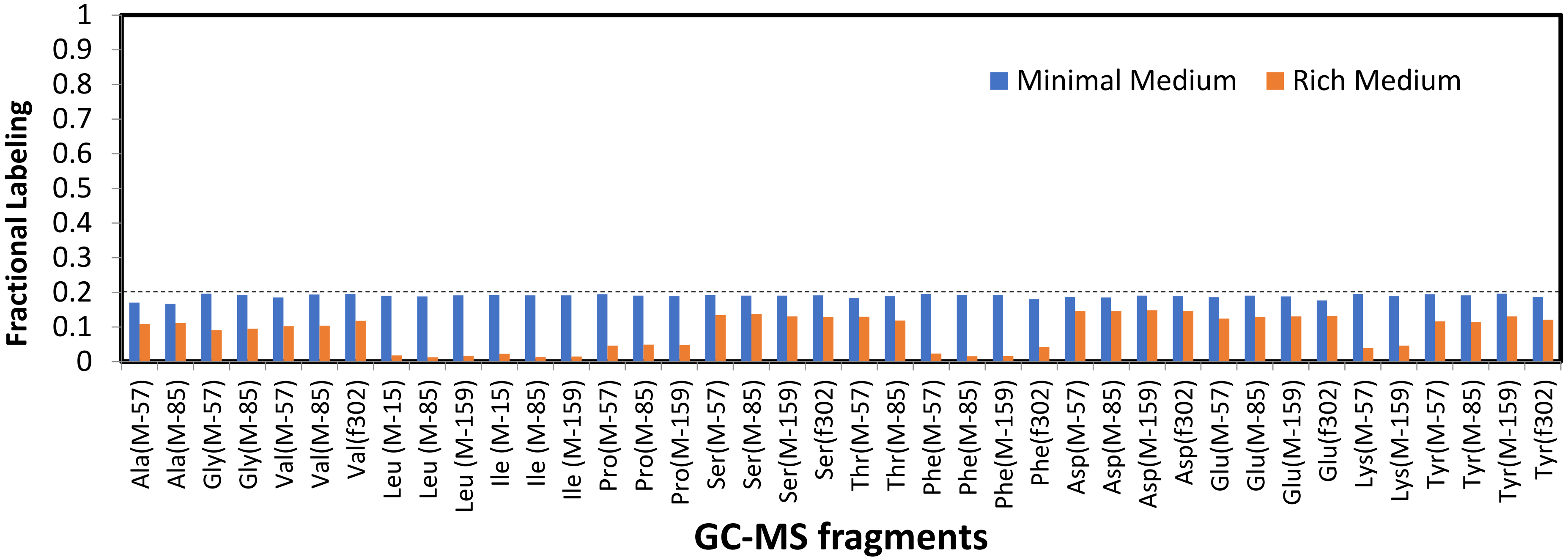

Figure S1 Fractional labeling of GC-MS fragments for *C. thermocellum* proteinogenic amino acids in CTFUD rich (red bar) and minimal medium (blue bar) by feeding 5 g/L 20% fully ^{13}C labeled and 80% fully unlabeled glucose. Dotted line represents the theoretical fractional labeling value of 0.2 when labeling steady state is reached.

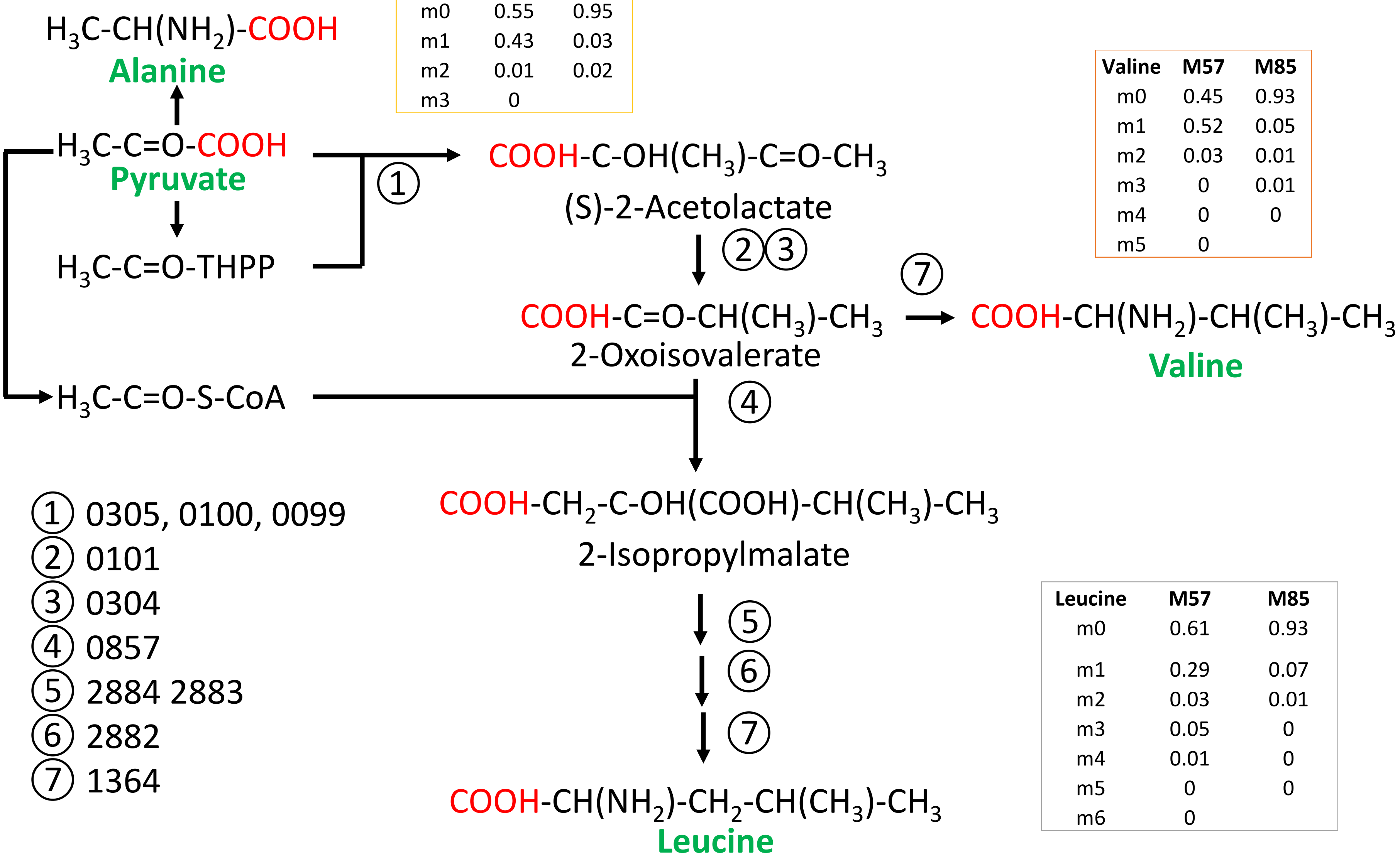

Alanine	M57	M85
m0	0.55	0.95
m1	0.43	0.03
m2	0.01	0.02
m3	0	

Valine	M57	M85
m0	0.45	0.93
m1	0.52	0.05
m2	0.03	0.01
m3	0	0.01
m4	0	0
m5	0	

Leucine	M57	M85
m0	0.61	0.93
m1	0.29	0.07
m2	0.03	0.01
m3	0.05	0
m4	0.01	0
m5	0	0
m6	0	

Figure S2 Isotope analysis using ^{13}C-bicarbonate confirms leucine and valine biosynthesis in *Clostridium thermocellum* DSM1313. ^{13}C-labeled positions are marked in red. ① acetolactate synthase (ALS), ②keto-acid reductoisomerase (KARI), ③ dihydroxy-acid dehydratase (DHAD), ④2-isopropylmalate synthase (IPMS), ⑤ 3-isopropylmalate dehydratase (IPMD), ⑥3-isopropylmalate dehydrogenase (IPMDH), ⑦ branched-chain aminotransferase (BCAT). These enzymes are encoded by corresponding genes in the genome of C. thermocellum DSM 1313. Their numbers are indicated. The mass distribution vectors for each amino acid in the pathway are presented. M57 and M85 represent two typical GC-MS fragments of the amino acids, respectively. M57 indicates the fragments lose a tert-butyl group from the derivatized reagent (Molecular weight 57). M85 indicates the one loses a tert-butyl group and a CO of the amino acid (Molecular Weight: 87). Therefore, M57 fragment reserves all carbons in the amino acid, while M85 loses the C1 carbon but reserves rest carbons in the amino acid.

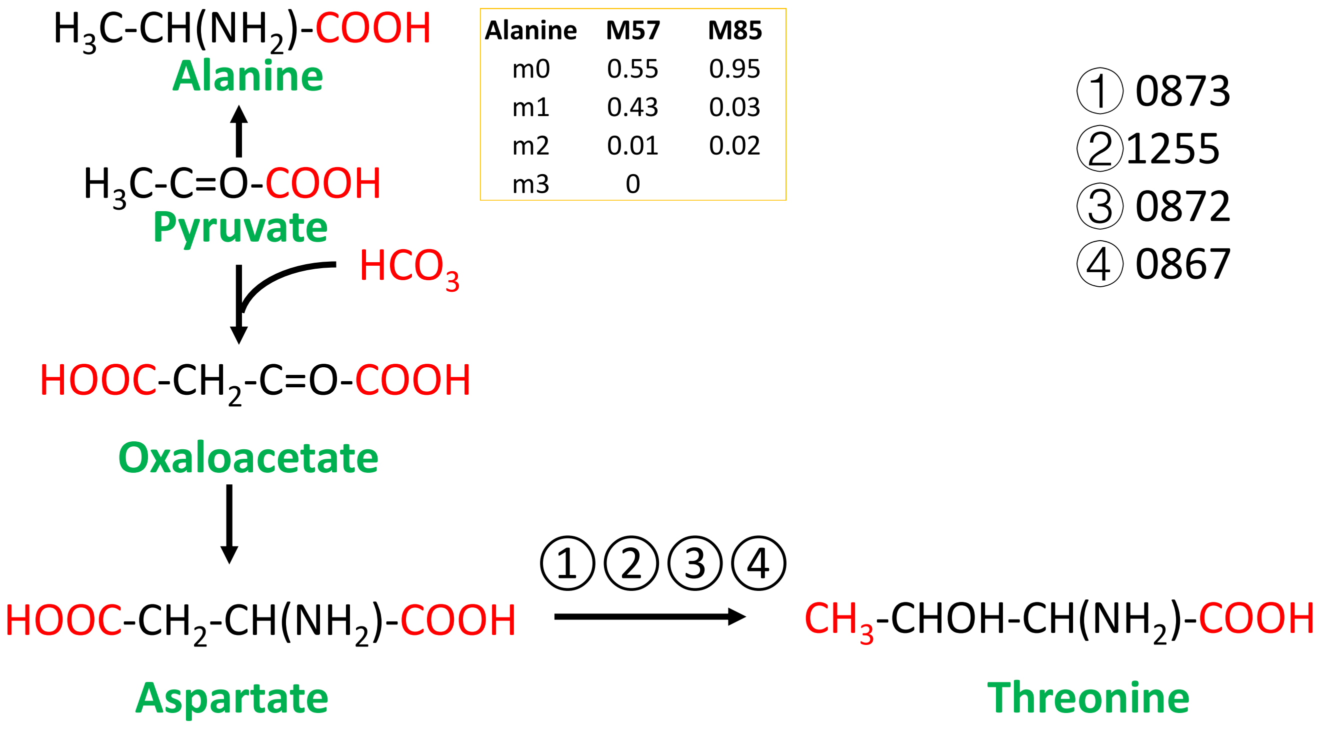

Alanine	M57	M85
m0	0.55	0.95
m1	0.43	0.03
m2	0.01	0.02
m3	0	

Aspartate	M57	M85
m0	0.34	0.46
m1	0.5	0.54
m2	0.15	0.01
m3	0	0
m4	0	

Threonine	M57	M85
m0	0.37	0.47
m1	0.5	0.51
m2	0.13	0.02
m3	0	0
m4	0	

Figure S3 Aspartate and threonine biosynthesis in *Clostridium thermocellum* DSM1313. ^{13}C-labeled positions are marked in red. ① aspartate kinase, ②aspartate-semialdehyde dehydrogenase, ③homoserine dehydrogenase, ④ threonine synthase.

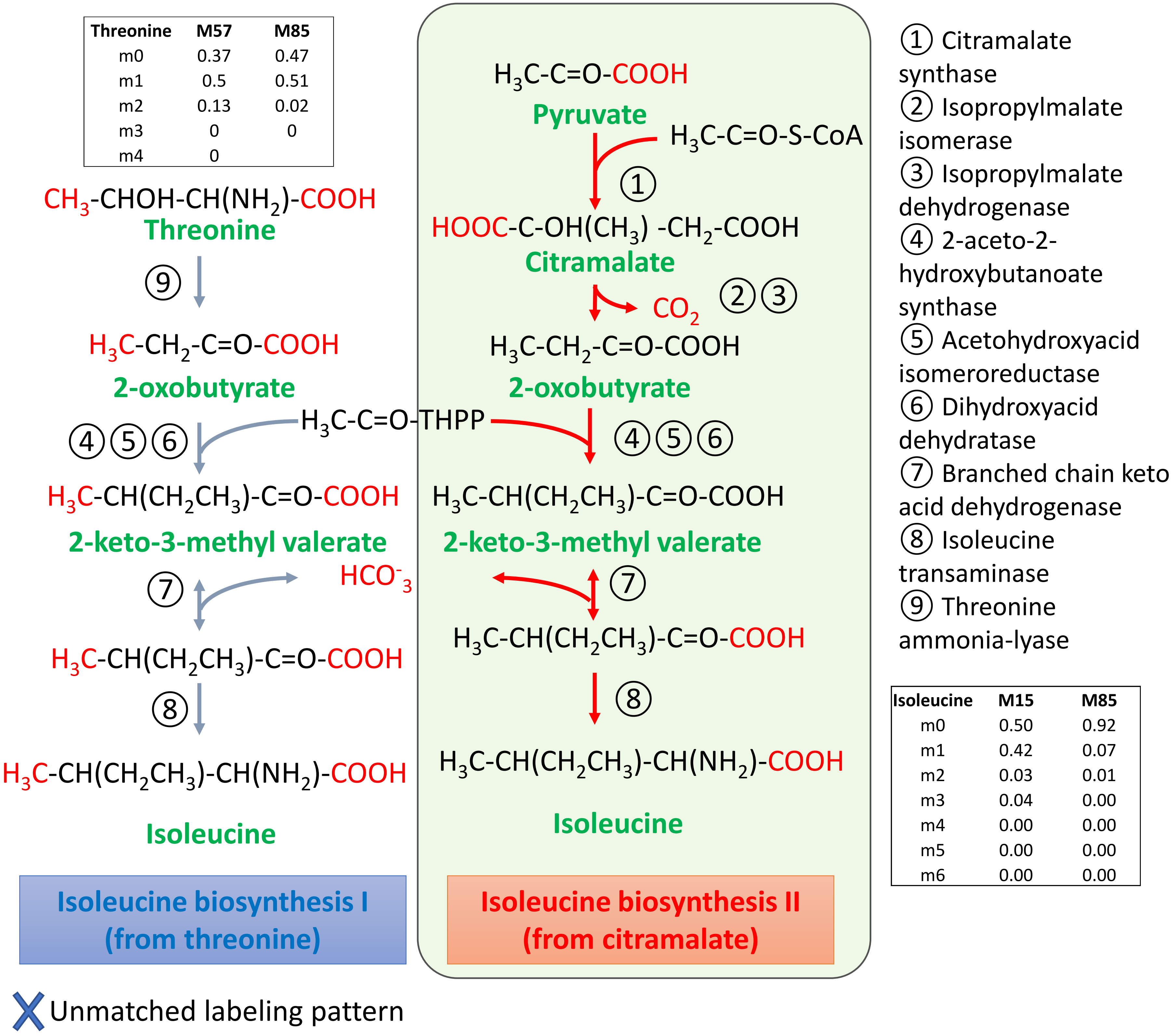

Threonine	M57	M85
m0	0.37	0.47
m1	0.5	0.51
m2	0.13	0.02
m3	0	0
m4	0	

Isoleucine	M15	M85
m0	0.50	0.92
m1	0.42	0.07
m2	0.03	0.01
m3	0.04	0.00
m4	0.00	0.00
m5	0.00	0.00
m6	0.00	0.00

Figure S4 Proposed isoleucine biosynthesis from threonine and citramalate pathway. (Labeling experiment used ^{13}C-bicarbonate as the carbon source.) ^{13}C-labeled positions are marked in red. The confirmed citramalate pathway is boxed.

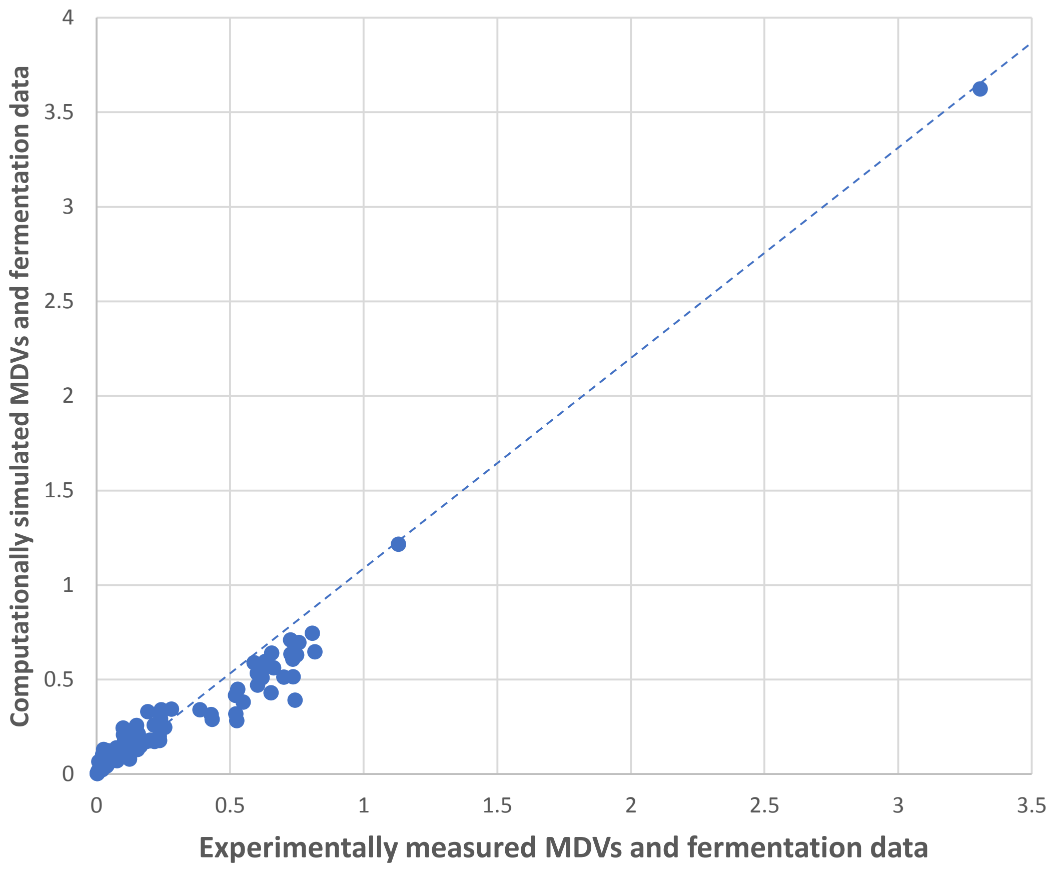

Figure S5. ^{13}C-MFA model fitting for *Clostridium thermocellum* metabolism. The data points include the mass isotopomer distributions (MID) of GC-MS fragments for amino acids including ALA232, ALA260, Gly218, Gly246, Val260, Val288, LEU274, ILE200, ILE274, SER288, SER362, SER390, THR376, THR404, PHE234, PHE302, PHE308, PHE336, ASP302, ASP316, ASP390, ASP418, GLU330, GLU404, GLU432, TYR302 and extracellular fluxes for sugar, lactate, acetate etc. (Amino acids are represented with their 3-letter abbreviations and the m/z of the unlabeled fragments are represented by following numbers.)The fit between simulated and measured data is good with R^2 = 0.95.
