# Supplementary material for: Isotope-Assisted Metabolite Analysis Sheds Light on Central Carbon Metabolism of a Model Cellulolytic Bacterium Clostridium thermocellum
Source: Front Microbiol. 2018 Aug 23;9:1947. doi: 10.3389/fmicb.2018.01947 (PMC6115520; doi:10.3389/fmicb.2018.01947)
Supplement: Supplementary file 3 [file Data_Sheet_2.PDF]

Table S1 Measured cell growth rate and extracellular fluxes. (Data from biological triplicates.)

**Metabolic fluxes (mmol/gDW/h)**	**Glucose unit**	3.31 ± 0.56
	**Lactate**	0.22 ± 0.07
	**Formate**	0.59 ± 0.17
	**Acetate**	1. 13 ± 0.10
	**Ethanol**	0.73 ± 0.47
**Specific Growth Rate (h^{-1})**		0.26 ± 0.01
**Carbon Balance**		94%
